# Progress of the Medical Sciences

**Published:** 1901-06

**Authors:** 


### Progress of the Medical Sciences.

### MEDICINE.

The etiology and treatment of **Acute Rheumatism** are subjects of perennial interest, and in which nothing approaching finality has yet been achieved. When we remember what was the usual history of an acute attack some thirty years ago, we may well rejoice that much more can be done to give relief to the patient than was formerly the case. Then the course of the disease was painful and protracted, and fortunate was the patient who, in spite of all treatment, escaped the perils of peri-endocarditis. Now the treatment is almost a matter of routine, and many of our hospital patients are well on the road to convalescence before even the first visit of the physician in charge. Although the treatment has made so good an advance, it is only recently that we have begun to recognise how much the clinical evidence tends in favour of the infectious origin of the disease. It is difficult to grasp the new ideas that such maladies as pneumonia, pulmonary tuberculosis, and acute rheumatism are always due to the presence of a *contagium vivum*, and must therefore be included in the list of contagious diseases. Dr. Cheadle sums up the evidence as regards rheumatism as follows:—

"The occasional epidemic prevalence, the variability of type, the incidence upon the young, the occurrence of tonsillitis, of endocarditis, of pleurisy, of pneumonia, of erythematous eruptions; the rapid anæmia, the tendency to capillary hemorrhage, and albuminuria; the implication of the joints, the relapses, the occasional supervention of hyperpyrexia, the nervous disturbance, the specific power of salicylic acid, are all suggestive of an infectious disease."[1]

The Milroy Lectures on the natural history and affinities of rheumatic fever [2] added much important and extensive research on the epidemiology and seasonal prevalence of the disease, and Dr. Newsholme concludes a recent paper on this subject as follows:—

"Direct infection from patient to patient is rare, if it occurs. It seems most feasible to regard rheumatic fever as essentially a soil disease, due to a saprophytic soil organism, which is 'drowned out' in wet years, multiplies rapidly in dry years, and is transferred to the human recipient by unknown means. Possibly dust convection accounts for a large proportion of the

[1] Quoted by Poynton, *Practitioner*, 1901, lxvi. 28.

[2] Newsholme, Milroy Lectures, abstract in *Brit. M.J.*, 1895, i. 527, 581.

cases. In dry years the number of contagious particles is probably increased, and the facility with which they are communicated to the human subject is similarly increased. In the light of our recently acquired knowledge of the methods of infection in malaria, it is not, perhaps, too far-fetched to surmise that inoculation of the contagion of rheumatic fever may be caused by domestic vermin, or that the house-fly may convey it to milk or other foods."[1]

Some new points in the pathology of rheumatic fever, considered as the result of infection by a diplococcus, have been recently given by Dr. F. J. Poynton.[2] He describes a diplococcus which has produced, on intravenous inoculation into rabbits, the clinical appearances of rheumatic fever. He, in conjunction with Dr. Alexander Paine, has isolated these organisms from the blood, urine, and tonsils of the living subject suffering from rheumatic fever, and also from the pericardial exudation and cardiac valves after death. They were demonstrated in the visceral and parietal pericardium, cardiac valves, the pleural and pericardial exudations, the tonsils, and rheumatic nodules. They have produced, on intravenous inoculation into rabbits, polyarthritis, valvulitis, pericarditis, pleurisy, pneumonia, chorea, and nodules. They also produced coagulation-necrosis in the kidneys and liver. From their investigations Drs. Poynton and Paine deduce the fact that this diplococcus is a cause of rheumatic fever. They add that "it is not easy to demonstrate the organisms in the human tissues. Apart from their small size, and the question of technique, there is the difficulty that rheumatic fever is rarely fatal when its lesions are in the earliest phases, and the organisms are rapidly destroyed in these lesions. . . . A study of their life history in the human and animal tissues leads to the conclusion that they are destroyed at the sites of the local lesions. . . . The clinical expression of this conclusion is that the disease usually runs a favourable course. . . . The rapid destruction of the organisms by the human tissues receives a clinical interpretation in the fleeting character of the polyarthritis and ephemeral character of many of the symptoms of rheumatic fever." The authors further think that they have found an explanation of the relapses which are so frequent: they found in the healing lesions, and in cultures on suitable media, a solitary coccal form, larger than the individual element of the diplococcus. This form persists after the disappearance of the diplococcus, and possibly may persist in the living tissues for some considerable period of time, and recover virulence if the vitality of the tissues be lowered by exposure to cold, &c.

Drs. Poynton and Paine[3] have further pointed out that "the presence of the organisms in that form of arthritis brought

[1] *Practitioner*, 1901, lxvi. 21.
[2] *Ibid.*, 22; also *Lancet*, 1901, i. 1260.
[3] *Lancet*, 1901, i. 858.

acute rheumatic affections of joints into line with arthritis the result of septic gonorrhœal and pneumococcic infections. According to the researches of Dr. G. A. Bannatyne, Dr. A. S. Wohlmann, and Dr. F. R. Blaxall, rheumatoid arthritis must be included in that group also. . . . Another point to which they wished to call attention was the condition of the kidneys in acute rheumatism. In fatal cases they had found very definite changes in the parenchyma, especially of the convoluted tubules. . . . Further, they had isolated the diplococcus from the urine in acute rheumatic pericarditis. . . . They had collected facts to show that some cases of malignant endocarditis were truly rheumatic and not the result of secondary infections."

Dr. C. O. Hawthorne, in discussing the relation of acute rheumatism to chronic rheumatoid arthritis, remarked that "one of the most confident grounds on which an endeavour had been made to mark a distinction between rheumatism and rheumatoid arthritis was the absence from the latter condition of cardiac disease. Endocarditis, they all recognised, was in the great majority of instances an evidence of rheumatism, but it was an expression of rheumatism which was specially prone to manifest itself in childhood and early adult life. It was by no means a common expression of rheumatism in middle life, but the majority of patients with chronic rheumatoid arthritis were those who were approaching or had reached middle age. Therefore it by no means followed that the absence of cardiac complications from the great majority of cases of rheumatoid arthritis proved that it was a condition quite distinct from rheumatism. Moreover, Charcot had stated that cardiac complications and valvular disease occurred fairly frequently in cases of rheumatoid arthritis."[1]

It is interesting to note that an infective arthritis due to the pneumococcus has frequently been observed since its first description by Weichselbaum in 1888. A paper by Dr. Edward J. Cave (Bath)[2] describes a typical case of the acute suppurative form of the disease occurring as a complication or immediate sequel of pneumonia. He points out that "the association of the arthritis with other localisations of pneumococcic infection is the rule. The associated lesion is commonly in a vital part, and threatens life more than the arthritis. . . . But often there has been a widespread infection of the system by the virus, with malignant endocarditis, pleurisy and empyema, pericarditis, nephritis, meningitis, and peritonitis."

Dr. G. A. Gibson considers it highly probable that all cases of acute and subacute cardiac diseases are of microbic origin, and all may therefore be termed infective.[3]

Cases illustrating the possible infectiousness of acute rheumatism have been reported recently.[4]

[1] *Ibid*, 859. [2] *Ibid.*, 82.
[3] *Practitioner*, 1901, lxvi. 46. [4] *Ibid.*, 79.

Although the evidence before us tends to indicate that the acute arthritis of acute rheumatism and that of rheumatoid arthritis may both be dependent upon the presence of micro-organisms, yet the organisms described differ in character and in life history. Dr. Bannatyne concludes that the two diseases in the acute stages are quite separate and distinct,[1] and he holds that the chronic variety of rheumatoid arthritis follows altogether a different course to the acute forms, and might well be secondary degenerations following on any acute joint trouble.

Dr. St. Clair Thomson[2] admits a general acceptation of the view that an undoubted association exists between rheumatism and tonsillitis. He states that "the tonsil has been well termed by Gerhardt a physiological wound—an opening into the system which, if not maintained in a healthy condition, may allow the passage of general infection. How true this may be is shown by Jessen, who has recorded four cases of serious general infection from the tonsils—acute articular rheumatism, acute pyæmia, streptococcal and staphylococcal pneumonia." The observations of Poynton and Paine carry this question a stage further, inasmuch as they were able to produce a non-suppurative valvulitis and pericarditis in the rabbit by injection of certain diplococci isolated from the throat during an attack of angina faucium in a patient with rheumatic fever.[3]

The *rôle* of the tonsils in association with acute rheumatism is suggested by the observations of F. Meyer,[4] who, in making bacteriological investigations in cases of acute articular rheumatism, having failed to obtain results from the direct examination of the joint exudate, made cultures from the tonsils, and discovered a diplococcus growing in chains which closely resembled the organism described by Wassermann. This organism was not found on the tonsils of persons who had not acute rheumatism. Injected into animals, it produced a sero-purulent exudation in the joints, and in about one-fourth of the animals there was a verrucose or ulcerative endocarditis.

It has long been generally admitted that an intimate association exists between rheumatism and chorea, and a paper by Dr. George F. Still[5] points out that "in the light of bacteriology chorea may prove, in some cases at least, to be directly or indirectly, the result of microbic infection, and therefore just as truly a manifestation of rheumatism as the articular symptoms. If this were so, chorea, like the heart affections and the rheumatic nodules, might be expected to precede the joint symptoms in some cases, and might be the first, or possibly the only, clinical manifestation of rheumatism." The rheumatic nodule has long been looked upon as an important

[1] *Lancet*, 1901, i. 936. [2] *Practitioner*, 1901, lxvi. 35.
[3] *Lancet*, 1900, ii. 861 *et seq.* [4] *Deutsche med. Wchnschr.*, Feb. 7, 1901.
[5] *Practitioner*, 1901, lxvi. 53.

manifestation of rheumatism, and Dr. Still observes that "the histological character of these nodules shows that they are exactly analogous to the fibrinous deposit on the inflamed endocardium or pericardium, and their association almost always with endocarditis suggests that they are due to the same cause, the rheumatic irritant whatever it be."

Drs. Poynton and Paine have recently demonstrated the diplococcus in three rheumatic nodules, they have isolated the diplococcus, and by intra-venous inoculation of this culture they have produced valvulitis, pericarditis, and polyarthritis in a rabbit. They have further isolated the diplococcus from the joint exudate of this rabbit.[1]

As regards the treatment of rheumatic fever, an excellent summary of what can be done has recently been given by Dr. Arthur P. Luff.[2] In spite of the moderate commendation of the salicylates given by so experienced a physician as Osler,[3] few will agree with him that "medicines have little or no control over the duration or course of the disease, which, like other self-limited affections, practically takes its own time to disappear." We agree with Dr. Luff "that the patient should be got under the influence of salicylates as soon as possible, that complete and prolonged rest should be enforced, that he should be maintained free from pain, that he should be kept on a fluid and mainly a milk diet for some time, and that free diuresis and a daily evacuation of the bowels should be secured." The contention of Maclagan that the salicylates and salicin exert a specific and curative action on rheumatic fever is borne out by almost universal experience. Given in sufficient quantity, they rarely fail to relieve the pain and reduce the temperature. It may well be contended that they destroy the alleged micro-organism of the disease. It is due to the fact that they so quickly give relief that they are often discontinued too soon, and thus follows the too frequent relapse. The nauseating taste of the salicylate is a difficulty in its administration, and the occurrence of deafness, tinnitus, and nausea often lead to their discontinuance when they are still required. Many substitutes have been tried, which, whilst devoid of the unpleasant by-effects of the salicylates, have also been for the most part less actively beneficial. A new preparation called Aspirin, a combination of acetic and salicylic acids, the acetic ester of salicylic acid, is said to possess the following advantages over the salicylates:—

1. It does not cause any irritation of the stomach or disturb digestion.
2. Does not cause tinnitus aurium.
3. Does not depress the heart's action.

From a limited experience of the drug, the writer is able to say that it appears to be as efficient as the salicylates, whilst it

[1] *Lancet*, 1901, i. 1261. [2] *Ibid.*, 64.
[3] *Principles and Practice of Medicine*, 3rd Ed., 1898, p. 174.

is less nauseating and less likely to give rise to unpleasant effects on the nervous system.

Dr. Caton's method of blistering along the course of the intercostal nerves for the prevention of valvular disease of the heart does not appear to receive that amount of attention which the importance of the subject deserves.[1]

R. Shingleton Smith.

### SURGERY.

It is now eight years since Dr. William White, of Philadelphia, proposed **castration as a remedy for prostatic hypertrophy,** and in the "Progress of the Medical Sciences" in this *Journal* for June, 1895, I gave an account of this method of treatment and a table of some of the recorded cases. Two years after the publication of Dr. White's original communication, he published a paper giving the results of the operation in 111 cases, and in 1896 Dr. Cabot tabulated 99 additional cases. Last year Dr. Wood, of Philadelphia, published[2] brief details of 159 more cases, and it, therefore, seems a suitable time to review the results of this operation, and also of vasectomy for the same disease. Taking Dr. White's 111 cases first, we find that the prostate atrophied in about 87 per cent., and return of the power of micturition occurred in 66 per cent., with relief or cure of cystitis in 52 per cent.; 18 per cent. of the cases died; but Dr. White thought that if cases were excluded in which the operation could not have been the cause of death, the mortality of the operation in this series of cases might be taken to be 7 per cent. Out of the 99 cases collected by Dr. Cabot only 66 were published with sufficient detail to be of any value, and of these 66 Dr. Cabot considered that 83 per cent. were much better for the operation. The mortality in his series was also 19 per cent., but no doubt only a few of these died from the effects of the operation. Dr. Wood has collected records of 159 cases of castration, not included in Dr. White's or Dr. Cabot's lists. Of these 130 are available for evidence as to the result of the operation; and in almost half of these cases it is definitely stated, and in many more it seems to be implied, that the prostate atrophied after the operation. The power of micturition was improved or wholly restored in many, and cystitis was relieved or cured in 18 per cent., and in some cases the cure of severe cystitis was a marked feature. The mortality did not exceed 8 per cent., and only a few of the deaths seem to have been due to the operation itself, whereas in several of the fatal cases improvement followed the operation. Probably most died from secondary kidney

[1] *Brit. M. J.*, 1900, i. 631, 765. [2] *Ann. Surg.*, 1900, xxxii. 316.

disease, which an earlier operation might perhaps have averted. Only in six cases were there any mental symptoms. The mental condition is called delirium in most of these. When we remember that the operation is nearly always performed after middle life, and often in the elderly, it seems to me very doubtful if mental symptoms are more common after castration than after any other operation requiring anæsthesia in old people. No change in the voice or alteration of disposition has been noted in any of the cases. In a few cases in which vesical calculus and hypertrophied prostate have been present, the stone has been crushed after atrophy of the prostate had followed castration or vasectomy; and in one case an attempt to get a sound into the bladder failed before, but succeeded after, the size of the prostate had been reduced by castration. Bruns, of Tübingen, collected records of twenty cases in which the catheter had been used from two to twenty years, and then had castration performed, and eight of these had return of the function of micturition and were able to dispense with the use of the catheter. It is clear that we cannot promise any patient return of the power of micturition, even if the prostate atrophies (as it almost certainly will) after castration, if he has had to draw his urine off by catheter for some considerable time, but power returns in many such cases. Again, the amount of residual urine may or may not decrease or disappear. It did so in 25 per cent. of Dr. Wood's cases. In one case of my own, although the prostatic overgrowth completely disappeared, the amount of residual urine remained the same.

It seems to me then that, with regard to the results of castration, we may conclude that the prostate will probably atrophy, and, therefore, if catheterisation is difficult and painful, it will become easy and painless. Even if no urine can be passed without the passage of the catheter, and the condition has persisted for some few years, the need for the use of the catheter may cease. The amount of residual urine may be much reduced or disappear, and the frequency of micturition much lessened. Cystitis may be greatly improved, and in many cases is cured as the obstruction disappears. Although many cases die after castration from cystitis and secondary renal infection or atrophy, and from the exhaustion of the frequent painful micturition disturbing their sleep, yet in very few cases indeed has castration been a cause of death, and the risks of this operation are not to be compared with those of intravesical prostatectomy.

Dr. Wood has collected records of 193 cases of **vasectomy for enlarged prostate.** Unfortunately the results are not given so fully as those of castration, and, therefore, it is difficult to judge as to the benefit of the operation in these cases. So far as the evidence goes it shows that it does not produce nearly so decided a decrease in the size of the prostate as castration, but in many cases there is an improvement in the power of micturition, diminution in the amount of the residual urine, and relief of cystitis. In 15 per cent. of the cases it is, Dr. Wood says, distinctly stated that no improvement followed the operation. There was nearly as large a percentage of deaths after the operation (6.7 per cent.) as in Dr. Wood's series of cases of castration, showing how little either operation has to do with the fatal termination; and the fact that some mental disturbance followed this operation of vasectomy in a few cases, also shows how little the actual removal of the testicles has to do with the mental symptoms following castration. It would seem then that the risks of castration are, if any, only very slightly greater than vasectomy, whereas the benefit is likely to be distinctly more marked after castration. But many patients will be willing to have vasectomy done, whereas they cannot bring themselves to entertain the idea of losing their testicles.

Since the new method of treating tetanus by intracerebral injections of the antitoxin has now been employed in at least forty-eight cases, it seems a fitting time to refer to a recent paper by Moschowitz,[1] giving a table of 290 cases of **tetanus treated by subcutaneous or intravenous injection** and forty-eight by intracerebral injection. The mortality of all the cases of tetanus treated in the London Hospital (without antitoxin) in sixteen years was found by Mr. Dean[2] to be 80 per cent.; and it has been stated by others that all the very acute cases die, and that 90 per cent. is the general mortality of the disease. The mortality in the 338 cases treated by antitoxin collected by Moschowitz was 42 per cent.; but it must be considerably higher than this, as many fatal cases would not be reported. This is not very encouraging, but it probably indicates that the antitoxin is quite worth employing. The results from intracerebral injections are not any better than the other method, though the experiments of Roux and Borrel[3] promised much for this method, for they saved thirty-five out of forty-five animals by its use, whereas only two were saved out of seventeen by the subcutaneous method. If the antitoxin is injected very slowly into the brain, and the head is kept very still so that the needle will not lacerate the brain, the injections seem to do no harm. The *post-mortem* examinations made in several cases failed to show any signs of gross cerebral injury. The injections are made into both frontal regions, and only quite small trephine openings are required. The method is based on the belief that the toxin of tetanus has a special affinity for the central nervous system, and that it would, therefore, be wise to get the antitoxin directly applied to it.

[1] *Ann. Surg.*, 1900, xxxii. 219. [2] *Brit. M. J.*, 1894, ii. 579.
[3] *Ann. de l'Inst. Pasteur*, 1898, xii. 225.

But how can we expect to get the diffusion of the antitoxin all over the brain and spinal cord by injecting it into the frontal lobes? Surely an injection into the sub-arachnoid fluid might be more likely to bring it quickly in contact with the higher centres. Experiments have indeed been made by injecting the antitoxin into the subdural space of the cord in animals inoculated with tetanus,[1] but without success.

The statistics collected by Moschowitz bear out the generally received view that the shorter the incubation period of tetanus the more severe the form of the disease. The author considers that it has been demonstrated that the cerebro-spinal fluid of tetanus patients is more toxic than the blood, and that the anterior horns of the spinal cord are specially affected by the toxin of the disease. He points out that although the use of the serum is a most important factor in the treatment of the disease, other measures should not be neglected, and he calls attention to the chemical agents generally regarded as most potent in the destruction of the bacilli and their spores in the wound—a very important element in the treatment—and recommends the use of 1 in 1000 perchloride of mercury solution with ½ per cent. hydrochloric acid.

Our knowledge of the **surgical treatment of pericardial effusion** has been extended by a recent paper by Dr. Porter, of Boston.[2] He has collected the records of seventy-five cases of operation for suppurative pericarditis. Twenty-four are referred to in a paper published in 1897,[3] and fifty-one are recorded in his more recent communication published last year.[4] He has made a careful study of the relation of the pleura to the pericardium with reference to deciding where the pericardium should be opened, and shows how close to the sternum the pleura may come, even as low as the sixth left interspace. After making experiments on the dead body, Porter decided to recommend the following method, because it will avoid opening the pleural cavity, and drainage will remain good after the sac has contracted. The fifth costal cartilage on the left side is resected, the division being made close to the sternum, the internal mammary artery and vein are double-ligatured and divided, and the pericardium incised, the edges stitched to the superficial structures, and a drainage tube introduced. Irrigation of the pericardial sac has been practised in more than half the cases, and is recommended by Porter with the object of removing fibrinous masses. Only in one case has any harm resulted from irrigation. In this instance the exit of fluid was obstructed by pieces of fibrin. He considers that the operation can be done in many cases under local anæsthesia. Porter is not the only surgeon who has

[1] *Berl. klin. Wchnschr.*, 1898, No. 49.

[2] *Ann. Surg.*, 1900, xxxii. 769.

[3] *Tr. Am. Surg. Ass.*, 1897, xv. 127. [4] *Loc. cit.*

recommended resection of the fifth left costal cartilage as the best method of reaching the pericardium. Ollier, Gussenbauer and Körte have all done so, and Mr. Betham Robinson adopted this method when operating on a child and found it satisfactory, though the child did not recover.[1] For a list of the many other situations in which the opening has been made, Porter's article must be referred to;[2] but one method of operating, lately proposed by Mr. Allingham,[3] must be noticed. Mr. Allingham records an unsuccessful case in which the method of drainage by removal of the fifth cartilage was employed, and proposes a method which he has tried twelve times on the dead body. The idea of the operation is, to reach the pericardium from below, at the most dependent part of the sac for drainage, and it is claimed for this method that the pleural cavity cannot possibly be injured. The seventh costal cartilage is exposed by an incision along its lower border, and the separation of the abdominal muscles from it. The cellular interval between the attachment of the diaphragm to this cartilage and to the xiphoid appendix is enlarged by cutting or tearing through the diaphragm as far as needful, and the loose fatty tissue being displaced the pericardium is reached. If the peritoneum is exposed it must be pushed down out of the way, just as it is in a suprapubic cystotomy

One advantage of avoiding the pleura in tapping the pericardium is shown by the fact that in two cases referred to by Jacobson in his work on *Operative Surgery*, the left pleural cavity was tapped under the impression that the pericardium had been reached, because the puncture had been made some distance from the left edge of the pleura, rather than close to that structure as recommended by Porter. With the statement of Porter's that because of the uncertain and varying relations of the pleura, and because of the anterior position of the heart in pericardial effusion, it is safer to make an open incision than to aspirate a serous effusion, we do not think most surgeons will agree.

It is perhaps hardly realised by some surgeons that **circular suture of the blood-vessels** has not only been shown to be possible by experiments on animals, but has now been successfully performed in a good many cases on men. In nine cases arteries have been successfully sutured, and it is stated[4] that no unsuccessful cases have been recorded; though this, of course, by no means shows that there have been no unsatisfactory results. Dr. Kuemmel, of Hamburg, has recorded[5] one case in which a portion of the femoral artery was resected with some malignant glands and the ends united. The circulation in the vessel was controlled with artery forceps

[1] *Brit. M. J.*, 1898, ii, 1605. [2] *Ann. Surg.*, 1900, xxxii. 774.
[3] *Lancet*, 1900, i. 693. [4] *Ann. Surg.*, 1900, xxxii. 730. [5] *Ibid.*, 728

covered with rubber tubes. The ends of the divided artery could only be approximated by extensive freeing of the vessel from its bed and flexion of the thigh. Circular suture was applied, and only penetrated the outer coat. The patient died a few months later, and at the *post-mortem* the line of suture in the vessel was not visible, but clot occupied the lumen of the vessel up to the iliac. It does not seem clear if the lumen was completely filled by this clot, but if so the vessel might as well have been ligated. The same surgeon also records a successful case of resection and circular suture of a portion of the femoral vein. Two cases of suture of wounds of large arteries—one the internal carotid injured in the removal of cancerous glands, and the other the brachial artery in an operation for traumatic aneurysm—are recorded by Doerfler as operated on by Barré.[1] In both primary union occurred, and in one case, at any rate, it is clear the lumen of the vessel was not obliterated. The author has experimentally determined that the presence of the thread in the lumen of the vessel does not interfere with its patency. As rigid asepsis is essential for success, the method must never be attempted in any infected wound. Indeed it seems to me that, although the results so far have been very encouraging, the force of the circulation is often so great that there must be considerable risk of hemorrhage in the case of sutured arteries.

C. A. Morton.

### OBSTETRICS.

The existence of **uterine fibromyomata** in women has one most important aspect which has not received, perhaps, that amount of attention which is its due, in view of the serious complications which may arise.

The presence of fibromyoma cannot but impair the fertility of the patient, and, moreover, should conception occur, may be the cause of imminent risk, both from the effects of the tumour on the reproductive process and from the effect of parturition or abortion on the tumour. Ample evidence, as might be expected, is forthcoming of the results of the presence of fibromyoma on conception; this is only to be expected when we consider how often, apart from any mechanical obstacle, the uterus affected is the subject of chronic inflammatory changes in its mucous membrane. At the same time, although the probability that the patient with fibromyoma of the uterus will become pregnant is lessened, yet a sufficiently large number of pregnancies occur to render the study of this complication of much practical value. Hofmeier[2] concludes from the observation of 150 cases that unmarried women do not

[1] *Ibid.*, 730. [2] *Am. J. Obst.*, 1901, xliii. 252.

suffer from this neoplasm more often than married women, having regard to the proportion between the numbers of the two classes. As regards the married, the proportion of sterile marriages among those who suffer from uterine fibroids is more than double that of all marriages—25 per cent. of marriages when the woman suffers from fibroids being sterile, against 11 per cent. of all marriages. Among 550 patients, 224 were nulliparous and 70 were uniparous, which appears to show, in Hofmeier's opinion, that the absence of pregnancy favours the growth of fibroid tumours. This, however, would appear to be capable of another interpretation. He is of opinion that the existence of these tumours has little effect on conception, and rarely interferes with the course of pregnancy. He also states that labour proceeds favourably with some attention, but that operation may subsequently be necessary. These last two views will not meet with general acceptance, and we shall show later that others consider fibroid tumours very serious complications of gestation and parturition, often needing severe radical operations.

Kreutzmann[1] reports two cases of fibroids complicating labour: in the first the patient nearly died of *post partum* hemorrhage; she had suffered much pain throughout her pregnancy; the tumours, two in number, were found to have almost disappeared later. In the second case miscarriage occurred in the fifth month; the patient had two tumours the size of cocoa-nuts. In a second pregnancy both tumours had diminished in size, but abortion occurred, followed by sapræmia, for which curetting had to be performed.

Bland Sutton[2] gives records of some interesting cases. In one a submucous fibroid the size of the fœtal head was expelled after delivery—it became septic and sloughed, the patient dying in consequence. No operation could be done, as the patient's condition was too bad. He quotes a case reported by Yeld[3] in which a sessile tumour, mistaken for the placenta, was removed by the blunt hook, the patient dying two hours later from shock. The tumour weighed 4½ pounds.

In another case a tumour was felt in the pelvis, but could be pushed up out of it. It was suspected that it was ovarian, and it was tapped, but no fluid evacuated. The patient was delivered with considerable difficulty; a few days later the tumour became septic, and five weeks after delivery a sloughing fibroid was expelled from the uterus. The patient ultimately recovered.

Champneys[4] collected quite a large number of cases showing that fibroids often complicate labour and frequently cause death.

Sutton sets down the dangers as follows: (1) The presence of the tumour often leads to impaction and abortion, when there

[1] *Ibid.*, 166. [2] *Lancet*, 1901, i. 452.

[3] *Brit. M. J.*, 1871, i. 586; quoted by Bland Sutton, *loc. cit.*

[4] *St. Barth. Hosp. Rep.*, 1877, xiii. 114; quoted by Bland Sutton, *loc. cit.*

is great liability to hemorrhage. (2) A submucous tumour may become septic and slough; occasionally it is expelled before the presenting part, but more usually is extruded subsequently. (3) A submucous tumour may become œdematous—the emptying of the uterus being followed by its inflammation, and leading to peritonitis or the formation of very vascular adhesions. (4) Occasionally a pedunculated submucous fibroid may become incarcerated in the pelvis and simulate an ovarian tumour.

One of the earliest signs in **pregnancy complicated with fibroids** is pain usually due to impaction.

In two cases, unsuspected pregnancy of four months duration was found after hysterectomy for fibroids.

In two others enucleation of the tumour was performed without disturbing pregnancy. In one a fibroid had become impacted under the pelvic brim on one side, and caused rotation of the fundus uteri to one side until it lay almost horizontal; in the other case the tumour had to be separated from the anterior wall of the uterus and bladder. Pregnancy was undisturbed in both. In six cases quoted by Sutton in which fibroids were removed during pregnancy by various operators one patient died; the remainder went to full term. Gemmell[1] reports a case in which several tumours were enucleated and the patient went to term.

Cases in which the tumour underwent necrotic changes after abortion are reported by Sutton[2] and Cole Baker.[3] In Sutton's case symptoms of peritonitis arose; hysterectomy was done and the patient recovered. In Cole Baker's case the uterus was retroverted and incarcerated, and after reduction fever occurred, followed by abortion and symptoms of peritonitis. Hysterectomy was followed by the patient's recovery.

Sutton[4] remarks on the danger caused by abortion in fibroids owing to the likelihood of necrotic changes occurring in the tumour unless the most rigid precautions to secure asepsis are successfully used.

Pain in these cases is usually due to impaction or torsion of the enlarging uterus, either of which will lead to abortion unless the tumour is dealt with surgically.

Spinelli[5] removed from the uterus of a woman four months pregnant a fibroid weighing 3½ kilogrammes, or 7¾ pounds. Abortion followed, but the patient made a good recovery. During a subsequent pregnancy vesical symptoms became severe, and, by vaginal cystotomy, were removed two large calculi formed around two ligatures which had found their way into the bladder.

In one of Sutton's cases, after the reduction of a retroverted fibroid uterus, two swellings appeared in the iliac fossa, and

[1] *Lancet*, 1900, ii. 1413. [2] *Loc. cit.* [3] *Dublin J. M. Sc.*, 1900, cx.

[4] *Loc. cit.* [5] *Am. J. Obst.*, 1901, xliii. 132.

acute pain was present. Cœliotomy and hysterectomy were performed: two fibroids—one anterior and one posterior—were present, which had caused axial rotation of the uterus through a quarter of a circle. The left ovary and tube were also removed.

Septic infection of the tumour, necrosis and peritonitis, may each and all arise from either premature or full-term delivery—either from septic uterine infection, manipulation, bruising or pressure of the fœtal parts. One recorded case, apart from pregnancy, died from peritoneal infection transmitted through the Fallopian tubes. Even should natural attempts at conservation produce sealing of the abdominal ostium of the tube, pyosalpinx is likely to result, so that the prospect for these patients is by no means bright, lying between pregnancy prematurely terminated exposing the patient to danger from sepsis, and that terminated at term having superadded those dangers produced by obstructed labour. The point for decision would then be the advisability of allowing the patient to go to full term, or at any rate leaving her until the period of viability of the child was reached; generally this would not appear to be advisable, and in the majority of cases this point has been reached by a patient in whose case the question of the possibility of obstruction had not been recognised. The safer plan would be to avoid delay and so render the patient safe from the risks attendant on abortion by early operation. In cases of obstruction the usual course has been to attempt to push the tumour out of the way, and then to deliver by forceps or version; or, if this cannot be effected, to deliver by Cæsarean section, followed by removal of the uterus.

This method of procedure is one which is not attended with great difficulty, since the pelvic attachments are lax and easily separated. The great enlargement of the blood-vessels, however, should be borne in mind, since this renders the possibility of their division by the ligature greater.

Out of nine cases operated on in this way by different operators, one died. In only three, however, was the operation undertaken late in pregnancy. Pushing up the tumour, and delivery by forceps or version, is not a very safe proceeding when the patient is in labour, since some amount of tearing of the tumour is extremely likely to occur, if not from instrumental interference, at any rate by the efforts of the uterus to expel the fœtus. Should, however, this procedure be adopted, a constant watch for dangerous symptoms must be kept during the puerperium, and should these arise, the tumour must be removed without delay. Myomectomy and hysterectomy have been quite safely carried out during the puerperal period.

Cragin [1] reports a case in which, after a tedious deliver y

[1] *Am. J. Obst.*, 1901, xliii. 236.

completed by forceps and attended by much hemorrhage, the temperature remained febrile for a month after delivery. Hysterectomy was performed, the uterus and tumour weighing 8 lbs. Necrotic changes in the tumour were well marked.

Dudley [1] relates a case in which, after delivery effected by forceps, the patient had some elevation of temperature. On the 10th day he removed sixteen fibroids from the uterine wall of various sizes: four were larger than an orange, the remainder varied from the size of a pea to that of a walnut. In removing one of them the uterine cavity was opened, and it was found that the uterine wall had been torn by the forceps, and that to this the rise of temperature was to be attributed.

Eustache [2] reports a successful case of total abdominal hysterectomy for labour obstructed for several days by a large fibromyoma. The operation was easy, and he considers that it should always be adopted in these cases.

Lloyd Roberts [3] reports a case in which Cæsarean section was performed on a woman in whom the pelvic cavity was blocked by a fibroid, which rendered delivery *per vias naturales* impossible, as it reduced the conjugate diameter to an inch and a half. The child did well and was living at the time of the report. The patient had, however, considerable rise of temperature, due to sepsis, and was treated with injections of strepto-antitoxin. She eventually recovered.

Macleod [4] reports a case in which abdominal section was undertaken for apparent retroversion of the gravid uterus, with partial impaction at term; adhesions preventing rectification, Cæsarean section was performed, and, in addition, a fibroid removed from the anterior uterine wall. Reduction proved impossible, even after the removal of the child, owing to dense adhesions. The patient recovered.

Of the dangers which arise during parturition, apart from obstruction, the cases quoted by Kreutzmann are typical instances. The chances of severe *post partum* hemorrhage are vastly increased, and the difficulty of successfully combating it also.

Another danger, a case brought to my own knowledge will illustrate. A patient was delivered at term and the placenta retained. Hemorrhage of a moderately severe nature occurred. The practitioner in attendance proceeded to remove the placenta by introducing the hand into the uterus, but found that it had partially escaped into the abdominal cavity through a rent in the uterine wall by the side of the tumour. The laceration had occurred without producing any well-marked symptoms. The placenta was extracted, but the patient died a few days later of general peritonitis. The practitioner was single-handed, in a remote country district, where help could not be obtained.

[1] *Ibid.*, 237 [2] *Ibid.*, 253. [3] *Lancet*, 1900, ii. 1016.
[4] *Brit. M. J.*, 1901, i. 141.

A large number of cases of operation are recorded, of which the foregoing are only a small selection. It now remains to see how the results of operation compare with those of natural delivery.

Natural delivery in these cases is by no means a safe termination, but, on the contrary, one attended with grave risk to both the mother and child.

Hirst [1] gives the following records, collected by different authors, as shewing the prognosis of natural delivery. Nauss and Süsserott have collected 372 cases, with a maternal mortality of nearly 50 per cent., and an infantile mortality of nearly 60 per cent. Lefour, whom he also quotes, gives the maternal mortality of 300 cases as varying from 25 to 55 per cent., and the infantile mortality as 77 per cent.

We have already seen from the results of operations by Sutton and others, quoted previously, that the maternal mortality is not high considering the difficulties of the condition. Hirst quotes Dsirne's statistics, which bear out this view entirely. Dsirne collected 135 cases of operation, for this complication of pregnancy and parturition. The mortality was 5·9 per cent., which is sufficiently low. Even allowing for a mortality of treble this percentage, we should still have a considerable margin, as against the results of the natural process with the various ways in which fatal complications may be caused. He also states that pregnancy is interrupted in about 20 per cent. of the cases operated on.

WALTER C. SWAYNE.

### Reviews of Books.

[1] *An American Text-Book of Obstetrics*, edited by Norris, part ii., 1896, 558.

